# Acute pancreatitis after intragastric balloon insertion: case report

**DOI:** 10.1093/jscr/rjad093

**Published:** 2023-03-07

**Authors:** Abdulmajeed Ali Alkhathami, Zuhair Babiker Ahmed, Abdullah Mohammed A Khushayl, Faiz Alsaffar, Abdullah M Alshahrani

**Affiliations:** College of Medicine, University of Bisha, Bisha, Saudi Arabia; Bariatric & Metabolic Surgery Unit, Department of General Surgery, King Abdullah Hospital, Bisha, Saudi Arabia; College of Medicine, University of Bisha, Bisha, Saudi Arabia; Bariatric & Metabolic Surgery Unit, Department of General Surgery, King Abdullah Hospital, Bisha, Saudi Arabia; Department of Family Medicine, College of Medicine, University of Bisha, Bisha, Saudi Arabia

**Keywords:** obesity, intragastric balloon, pancreatitis, complications, Saudi Arabia

## Abstract

The intragastric balloon (IGB) is a relatively recent non-surgical weight loss technique that is now widely used in the world to treat obesity. However, IGB causes a wide range of adverse effects that range from minor ones, such as nausea, stomach pain and gastroesophageal reflux, to serious ones, such as ulceration, perforation, intestinal blockage and compression of adjusting structures. A 22-year-old Saudi woman presented to the emergency department (ED) with a history of upper abdominal pain that started 1 day before admission. The patient’s surgical background was unremarkable, and no other obvious pancreatitis risk factors were present. The patient underwent a minimally invasive treatment after being diagnosed with obesity (class 1), in which an IGB was inserted one and a half months prior to her ED presentation. She consequently began to lose weight (around 3 kg). The hypothesis states that pancreatitis following IGB insertion can be caused either by stomach distention and pancreatic compression at the tail or body or by ampulla obstruction due to balloon catheter migration at the duodenum. Heavy meal consumption, which may cause an increase in pancreatic compression, is another potential cause of pancreatitis in such patients. We believe that the IGB-induced compression of the pancreas at its tail or body was the likely cause of pancreatitis in our case. This case was reported because it is the first one from our city as far as we know. A few cases from Saudi Arabia have also been reported, and reporting them will help to improve doctors’ awareness of this complication, which can cause pancreatitis symptoms to be mistaken for something else because of the balloon-related effects on gastric distention.

## INTRODUCTION

One of the main public health issues facing modern society, which is also a huge global concern, is obesity. It is a known risk factor for many life-threatening diseases, including metabolic syndrome [1]. The most common way to lose weight is through bariatric surgery, although some people need non-surgical methods because of their contraindications to surgery. The intragastric balloon (IGB) is a relatively recent non-surgical weight loss technique that is now widely used in the world to treat obesity [2]. However, IGB produces a wide range of adverse effects that range from minor ones, such as nausea, stomach pain and gastroesophageal reflux, to serious ones, such as ulceration, perforation, intestinal blockage and compression of adjusting structures. According to the literature, a smattering of cases have reported acute pancreatitis as a complication of IGB placement [3]. As pancreatitis is a rare complication of IGB placement, to our knowledge, the prevalence of such a complication after IGB insertion has never been reported in any previous studies.

## CASE REPORT

The 22-year-old Saudi female in this case was single with no previous medical history. She presented to the emergency department (ED) with a history of upper abdominal pain that started 1 day before admission. The pain was continuous, started gradually and became progressive in nature with a severity of 8/10 with no diurnal variation or radiation after which the pain involved the entire abdomen. Moreover, it was sharp in nature, aggravated by movement and relieved slightly by rest. The pain was associated with nausea and vomiting with bile-free gastric content containing blood. No history of change in bowel habits, urine or cardiopulmonary symptoms was reported. The patient did not smoke or consume alcohol. Her menstrual cycle was regular with a normal amount of bleeding. The patient denied any history of abdominal trauma, scorpion bites, current medication use or previous similar complaints. Family history was insignificant for similar conditions and autoimmune or inherited diseases.

The patient’s surgical history was unremarkable, and no other clear risk factors for pancreatitis were found. The patient was known to have obesity (class 1) with a body mass index (BMI) of 33 kg/m^2^ for which she underwent a minimally invasive procedure, in which an IGB was inserted through an endoscope one and half months ago. She consequently began to lose weight (around 3 kg).

On examination, the patient presented with temperature of 38.3°C, heart rate of 112 bpm and blood pressure of 132/84 mmHg. The abdominal examination revealed bulging of the abdominal wall with diffuse pain and tenderness and sluggish bowel sounds.

Results from her blood/laboratory workup are shown in Table 1.

**Table 1:** The result of the patient’s blood workup on admission

Parameter	Patient result	Normal value
Serum creatinine	17 μmol/l	44–80 μmol/l
Serum amylase	294 U/l	28–100 U/l
Serum lipase	459.30 U/l	13–60 U/l
WBC	12.30 × 10^3^/μl	4–11 × 10^3^μ/l
Neutrophil level	83.80%	50–70%
Total bilirubin	8.90 μmol/	0–17.1 μmol/l
Alp	60 U/l	35–135 U/l
GGT	11 U/l	536 U/l
ALT	6.30 U/l	0–31 U/l
AST	10.90 U/l	0–32 U/l
Triglyceride	1.3 mmol/l	<1.7 mmol/l
Pregnancy test	Negative	

Alp: alkaline phosphate; GGT: gamma-glutamic transferase; ALT: alanine aminotransferase; AST: aspartame aminotransferase.

Ultrasound showed minimal free fluid at the left hypochondrium. Gall bladder was not tender on probing and normal in size and wall thickness. Her liver was average size with a normal caliber common bile duct and portal vein.

**Figure 1:**
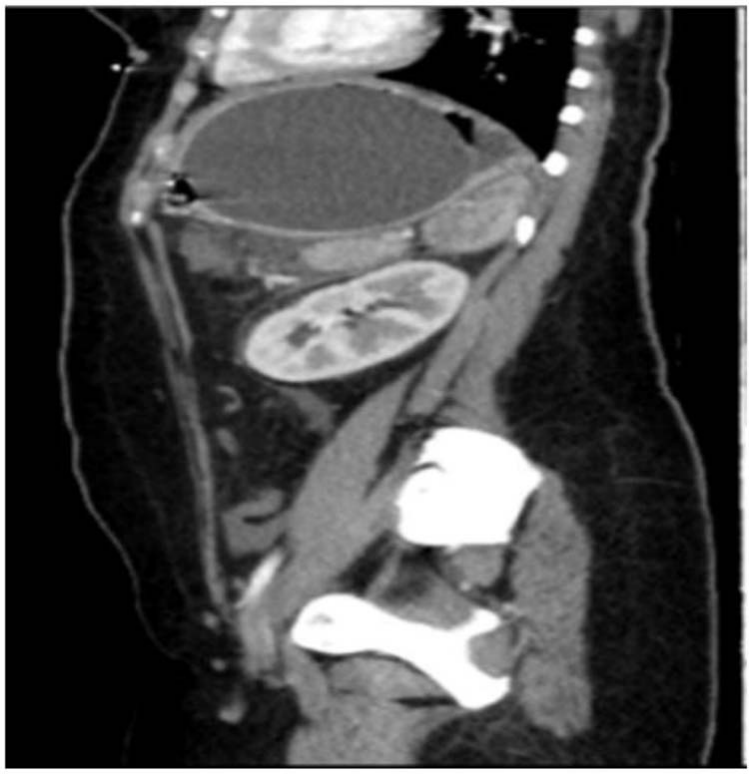
Axial computed tomography (CT) showing IGB compressing the pancreatic body with mild pancreatic edema, regional fat stranding and surrounding fluid collection seen tracking to left perirenal space.

**Figure 2:**
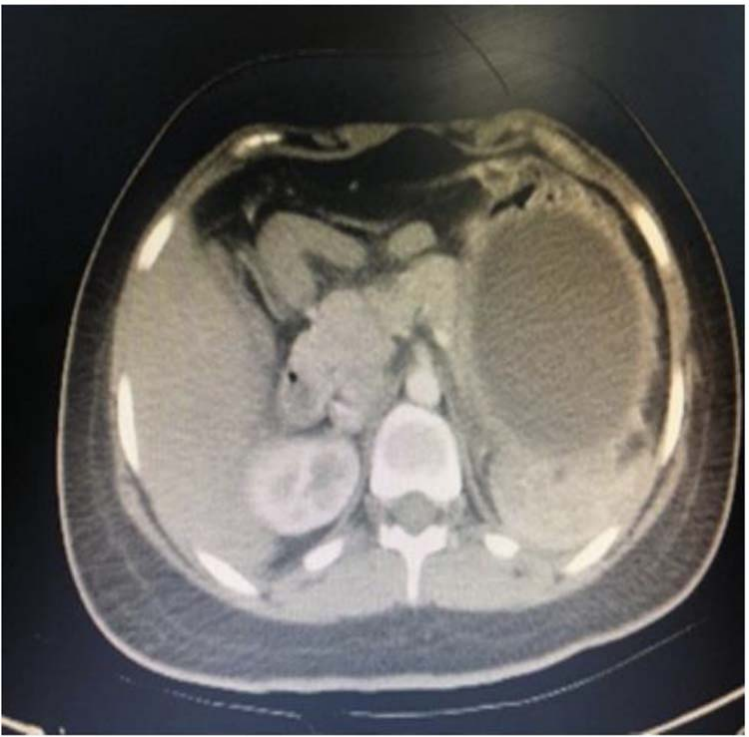
Coronal CT showing the position of the IGB.

A computed tomography scan with contrast of the abdomen showed gastric fundus distended by a well-defined structure (the balloon) seen compressing the pancreatic body with mild pancreatic tail edema, regional fat stranding and surrounding fluid collection seen tracking to the left perirenal space as shown in Figs 1 and 2. The liver was average in size with no gross contour irregularity or parenchymal abnormal attenuation. No hepatic focal lesion could be detected. Also, no evidence of intra- or extra-hepatic biliary radicle dilatation was detected. A patent average caliber of the portal vein was found, and no porta hepatis lymph nodes seen. No other abnormalities were detected. A magnetic resonance imaging scan was planned but could not be done because the patient had orthodontics (metallic object).

The patient was admitted as a case of non-biliary pancreatitis that we thought was due to the balloon compression on the pancreas. Some previous cases were found that supported our idea, so we discussed the findings with the patient and her family and they agreed to remove the balloon. The patient received nothing by mouth but had intravenous fluid and was treated with proper analgesia, then referred back to her attending gastroenterologist, who removed the balloon by endoscopy. The inner layer of the stomach was normal based on the upper endoscopy (no gastritis). After removal of the balloon, the patient’s symptoms improved, and she was discharged from the hospital and given an appointment for follow-up to look for pancreatitis-related complications.

## DISCUSSION

Morbid obesity can be treated in a variety of ways, from non-surgical to surgical methods. Diet, exercise, behavioral therapy and pharmacological therapy are examples of non-surgical procedures. A few endoscopic treatments, such as IGB placement, are also available. Surgical procedures include Roux-en-Y gastric bypass, band placement and sleeve. Although balloon placement for weight loss has been used since 1985, its clinical effectiveness has not been thoroughly evaluated [4]. The IGB is a saline-filled silicone bag that functions as a compartment to take up space and reduce the amount of stomach space that can be used to hold food. This process causes an early satiety and feeling of fullness. IGB is utilized as a non-operative technique in the management of bariatric patients and is gaining popularity because it offers a less intrusive and generally safe way to achieve rapid weight loss. A gastric ulcer may form as a result of the increased pressure that is placed on the stomach wall [5]. Pancreatitis related to IGB placement is a rare complication but has been reported. According to a study by Gore *et al*. [6], ‘patients can be at risk of pancreatitis anytime between 1 day and 11 months following insertion’. The placement of an IGB is a temporary procedure that was developed to help patients lose weight. Balloons should be removed at the end of a period that should not last longer than 6 months. If necessary, they can be replaced with a new one. Balloons remaining in the stomach for more than 6 months can lead to an increase in the possibility of complications [4]. In the present case, however, the patient developed complications only one and a half months after placement.

The general hypothesis suggests that such causes of pancreatitis after insertion of an IGB can be either due to stomach distention and compression of the pancreas at the tail or body or can result from obstruction of the ampulla due to migration of the balloon catheter at the duodenum [4, 7]. Another possible cause of pancreatitis in patients is heavy meal intake, which might have increased pancreatic compression. We assume that the probable cause of pancreatitis in our patients was attributed to compression of the pancreas at the tail or body by the IGB.

In conclusion, IGB is a relatively recent and effective non-surgical weight loss technique that is now widely used in the world for the management of patients who have not been successful in losing weight using conventional weight loss techniques.

This case was reported because, to the best of our knowledge, it is the first one from our city to be reported. A few cases from Saudi Arabia have been previously reported, and reporting them will help to improve doctors’ perceptions of this complication, which can present as pancreatitis symptoms but be misdiagnosed because of the balloon’s effect on gastric distention.

## Data Availability

The data that support the findings of this study are available from the corresponding author, A.A. Alkhathami, upon reasonable request.
