# Correction to: Acute pancreatitis after intragastric balloon insertion: case report

**DOI:** 10.1093/jscr/rjad265

**Published:** 2023-05-10

This is a correction to: Abdulmajeed Ali Alkhathami, Zuhair Babiker Ahmed, Abdullah Mohammed A. Khushayl, Faiz Alsaffar, Abdullah M. Alshahrani, Acute pancreatitis after intragastric balloon insertion: case report, *Journal of Surgical Case Reports*, Volume 2023, Issue 3, March 2023, rjad093, https://doi.org/10.1093/jscr/rjad093

In the originally published version of this manuscript, Abdullah Mohammed A. Khushayl's name was incorrectly written as Abdullah M. Alkhushayl.

This error has now been corrected.

